# Aspirin Eugenol Ester Reduces H_2_O_2_-Induced Oxidative Stress of HUVECs via Mitochondria-Lysosome Axis

**DOI:** 10.1155/2019/8098135

**Published:** 2019-09-09

**Authors:** Mei-Zhou Huang, Ya-Jun Yang, Xi-Wang Liu, Zhe Qin, Jian-Yong Li

**Affiliations:** Key Lab of New Animal Drug Project of Gansu Province, Key Lab of Veterinary Pharmaceutical Development of Ministry of Agriculture, Lanzhou Institute of Husbandry and Pharmaceutical Sciences of CAAS, Lanzhou 730050, China

## Abstract

The oxidative stress of vessel endothelium is a major risk factor of cardiovascular disorders. Antioxidative stress drugs are widely used in cardiovascular therapy. Aspirin eugenol ester (AEE) is a new pharmaceutical compound synthesized by esterification reaction of aspirin with eugenols and possesses antioxidative activity. The present study was designed to investigate the mechanism how AEE protects human umbilical vein endothelial cells (HUVECs) from H_2_O_2_-induced oxidative stress. H_2_O_2_ was given to the HUVECs with or without AEE pretreatment. Changes in the oxidative stress-related factors, including those related to the mitochondria-lysosome axis, were determined with Western blotting, cellular immunofluorescence, and enzyme activity test. The results showed that, in the HUVECs, 300 *μ*M H_2_O_2_ treatment significantly increased the apoptosis rate, MDA concentration, reactive oxygen species (ROS) production, mitochondrial membrane potential, expression of Bax and mature cathepsin D (CTSD), and activity of CTSD and Caspase3 (Cas3) but decreased the expression of Bcl2 and lysosomal membrane stability, while in the HUVECs pretreated with AEE, the above changes caused by either the stimulatory or the inhibitory effect of H_2_O_2_ on the relevant factors were significantly reduced. AEE pretreatment significantly enhanced the activity of cellular superoxide dismutase and glutathione peroxidase in the HUVECs. Our findings suggest that AEE effectively reduced H_2_O_2_-induced oxidative stress in the HUVECs via mitochondria-lysosome axis.

## 1. Introduction

The most global deaths and disability-adjusted life year events are associated with the cardiovascular diseases [1–3]. Endothelial dysfunction is a key weathercock of cardiovascular diseases [4–6]. Investigation of the incentives and mechanism of endothelial dysfunction and development of new drugs against endothelial dysfunction benefits the prevention and treatment of cardiovascular diseases. In recent years, it has been believed that oxidative stress is a key risk factor of endothelial dysfunction [7–11]. The drugs and active compounds with antioxidative activity tend to be the potential agents for the prevention of cardiovascular diseases [12–14]. Low concentrations of eugenol are known to act as an antioxidant and anti-inflammatory agent [15–18], but the instability and cytotoxicity of eugenol limit its application in antioxidative treatment [19–21]. It has been reported that low dose of aspirin protects the cell from oxidative stress [22–24]. Aspirin eugenol ester (AEE) is a new pharmaceutical compound synthesized by esterification reaction between aspirin and eugenols [25]. Previous studies have shown that AEE possesses desired antioxidant pharmacological activity and effectively reduced the side effect of both eugenol and aspirin [26, 27]. Moreover, AEE could reduce the H_2_O_2_-induced dysfunctions of NO in the vascular endothelial cells by enhancing the antioxidant capacity of cells [27]. Oxidative stress is an important factor causing dysfunctions of NO. To clarify the biological mechanism responsible for antioxidative effect will be helpful to further understand the effect of AEE on the H_2_O_2_-induced dysfunctions of NO. It has been shown that mitochondria play essential roles in oxidative balance of the cell [28, 29]. Lysosome regulates the cellular redox status by altering the autophagy degradation pathway, inhibiting lysosome enzyme function and reducing lysosome membrane damage [30, 31]. Mitochondria and lysosome regulate the cellular redox status interdependently [29, 32]. In the present study, the mechanism how AEE protects cell from oxidative stress was examined via mitochondria-lysosome axis. This will help in understanding the effect of AEE on cardiovascular diseases.

## 2. Materials and Methods

### 2.1. Chemicals

AEE transparent crystal with the purity of 99.5% by RE-HPLC was prepared in Lanzhou Institute of Husbandry and Pharmaceutical Sciences of CAAS. H_2_O_2_ solution (cat number: 323381), dimethyl sulfoxide (DMSO), reactive oxygen species assay kit, and trypsin-EDTA were supplied by Sigma (St. Louis, MO). Cell Counting Kit-8 (CCK-8) was from MedChemExpress (NJ, USA), DMEM/F12 (1 : 1), and fetal bovine serum was from Gibco (NY, USA). LysoTracker Red probe, Hoechst 33342 staining solution, cellular glutathione peroxidase assay kit, Cu/Zn-SOD and Mn-SOD assay kit, and mitochondrial membrane potential assay kit were purchased from Beyotime (Shanghai, China). MitoTracker Red CMXRos probe was from Thermo Scientific (MA, USA). Anti-Bid cleavage site, Anti-Bax, Anti-Bcl2, Anti-Bcl-XL, anti-Caspase3 (Cas3), anti-cytochrome C, and anti-Cathepsin D (CTSD) were purchased from Abcam (MA, USA) and the CTSD activity assay kit was from BioVision (CA, USA). An Annexin V/FITC apoptosis detection kit was from BD Biosciences (NY, USA). A Caspase-3 activity assay kit was from Cell Signaling Technology (MA, USA).

### 2.2. Cell Cultures and Cell Treatment

Human umbilical vein endothelial cells (ATCC® CRL-4053™) were purchased from ATCC (Rockville, MD). The cells were cultured in the cell culture flasks in DMEM/F12 (1 : 1) with 10% fetal bovine serum. Media were replaced every two days. Subcultures were performed with the trypsin-EDTA method. Experiments were subsequently conducted on 6-7 passages of cells.

HUVECs were randomly divided into 3 groups (*n* = 6): normal group, model group, and AEE pretreatment group. Cells in the normal group were incubated with the normal growth conditions. Those in the model group were incubated with the medium containing 300 *μ*M of H_2_O_2_ for 6 h. In the AEE pretreatment groups, the cells were cultured with the medium containing different concentrations of AEE (0.5, 1, 2.0, and 4.0 *μ*M) for 24 h before they were incubated with medium containing 300 *μ*M H_2_O_2_ for 6 h.

### 2.3. Apoptosis Detection with Flow Cytometry

In the HUVECs, the apoptosis induced by H_2_O_2_ was detected quantitatively with Annexin V/FITC apoptosis detection kit and flow cytometry. Briefly, the cells were collected and washed three times with cold PBS. Then, they were incubated and stained with FITC-Annexin V and PI at room temperature for 15 min in the dark. The double stained cells were analyzed using flow cytometry, and the following controls were used to set up compensation and quadrants: unstained cells, cells stained with FITC-Annexin V, and cells stained with PI.

### 2.4. Intracellular and Mitochondrial ROS Measurement

Intracellular ROS was measured with reactive oxygen species assay kits following the manufacturer's instructions. Briefly, the HUVECs were cultured on the 24-well glass bottom cell culture plate and treated with AEE and H_2_O_2_. Then, the cells were incubated with the ROS detection work solutions (10 *μ*M) at 37°C for 20 min in the dark. The relative fluorescence intensity of the cells was measured using a laser scanning confocal microscope (ZEISS LSM-800, Jena, Germany).

The mitochondrial ROS was detected by a MitoSOX probe following the manufacturer's instructions. Briefly, the HUVECs were cultured on the 24-well glass bottom cell culture plate and treated with AEE and H_2_O_2_. Then, the cells were incubated with the MitoSOX probe work solutions (4 *μ*M) at 37°C for 10 min in the dark. The relative fluorescence intensity of the cells was measured using a laser scanning confocal microscope (ZEISS LSM-800, Jena, Germany).

### 2.5. Malondialdehyde (MDA), GSH/GSSG Ratio, and Activity of Sodium Oxide Dismutase (SOD) and Glutathione Peroxidase (GSH-Px) Assay

The concentrations of MDA, GSH/GSSG ratio, SOD, and glutathione peroxidase activity in the HUVECs were determined using the commercial kits according to the manufacturer's protocols (see Section 2.1 for details).

### 2.6. Measurement of Mitochondrial Membrane Potential

A mitochondrial membrane potential assay kit was used to determine the mitochondrial membrane potential. Briefly, the HUVECs were cultured on the 24-well glass bottom cell culture plate and treated with AEE and H_2_O_2_. Then, the cells were incubated with JC-1 work solutions (5 *μ*M) at 37°C for 20 min in the dark. The relative fluorescence intensity of the cells was measured using a laser scanning confocal microscope (ZEISS LSM-800, Jena, Germany) with the positive control, in which the HUVECs were treated with CCCP (10 *μ*M) at 37°C for 20 min, then incubated with JC-1 work solutions (5 *μ*M) at 37°C for 20 min in the dark.

### 2.7. Lysosomal Membrane Stability Assay

Lysosomal membrane stability was determined with a LysoTracker Red probe. Briefly, the HUVECs were cultured on the 24-well glass bottom cell culture plate and treated with AEE and H_2_O_2_. Then, the cells were incubated with LysoTracker Red work solutions (10 nM) at 37°C for 40 min in the dark. The relative fluorescence intensity of the cells was measured using a laser scanning confocal microscope (ZEISS LSM-800, Jena, Germany). With the positive control, the HUVECs were treated with chloroquine (50 *μ*M), a lysosomal stabilizer, at 37°C for 24 h, then incubated with LysoTracker Red work solutions (10 nM) at 37°C for 40 min in the dark.

### 2.8. Cathepsin D (CTSD) Activity Assay

CTSD activity in the HUVECs was determined with an enzymatic assay method using a commercial kit according to the manufacturer's protocol (see Section 2.1 for details).

### 2.9. Protein Expression Analysis

The expression of Bid, Bcl2, Bcl-XL, Cas3, and CTSD was evaluated by Western blot analysis. In brief, the total protein of the HUVECs was isolated with RIPA lysis buffer. The concentration was quantified using the BCA method. SDS-PAGE (10%) electrophoresis and transfer of the separated proteins onto polyvinylidene fluoride membrane (Merck Millipore) were performed using standard procedures. The blots were incubated with primary antibodies against Bid, Bcl2, Bcl-XL, Cas3, CTSD, and internal control *β*-actin, and then incubated with horseradish peroxidase-conjugated secondary antibody. The results were observed using the Bio-Rad ChemiDoc™ MP imaging system and normalized to the corresponding internal control of *β*-actin for eliminating the variation of the total protein.

The expression of cytochrome C and Bax was evaluated with immunofluorescence. Briefly, the cells were cultured on glass coverslips in the medium and the mitochondria were labeled with the CMXRos probe; then, immunofluorescence analyzes 4% paraformaldehyde–fixed. 0.1% Triton X-100 permeabilized the cells labeling the primary antibody against cytochrome C and Bax, and then, they were incubated with the Alexa Fluor®-conjugated secondary antibody. The relative fluorescence intensity of the cells was measured using a laser scanning confocal microscope (ZEISS LSM-800, Jena, Germany). The colocalization data of cytochrome c and mitochondria have been acquired by ZEN blue software (ZEISS, Jena, German) following with the colocalization analysis.

### 2.10. The Activity of Cas3 Assay

Cas3 activity in the HUVECs was determined with the enzymatic assay method using the Cas3 assay kit according to the manufacturer's protocol.

### 2.11. Transfection of HUVECs with Lentivirus

The lentivirus labeled ubiquitin IRES-puromycin containing short interference (si) RNA oligonucleotides against Bcl2 (siBcl2) or control siRNA was used to transfect HUVECs for knockdown experiments, and the HUVECs were transduced with a lentivirus expressing Bcl2 gene labeled ubiquitin IRES-puromycin for overexpression experiments. The positive cells were selected using puromycin.

### 2.12. Statistical Analysis

All experiments and data analyses were carried out according to the blinding principles. Statistical analysis was performed using the SAS 9.2 (SAS Institute Inc., NC, USA). Where applicable, the values from treatment groups were normalized to the corresponding control values. All data were presented as means ± SD. The differences between groups were analyzed via one-way ANOVA followed with Duncan's multiple comparisons. Statistical significance was considered at *p* < 0.05.

## 3. Results

### 3.1. AEE Attenuated H_2_O_2_-Induced Apoptosis in HUVECs

**Figure 1:**
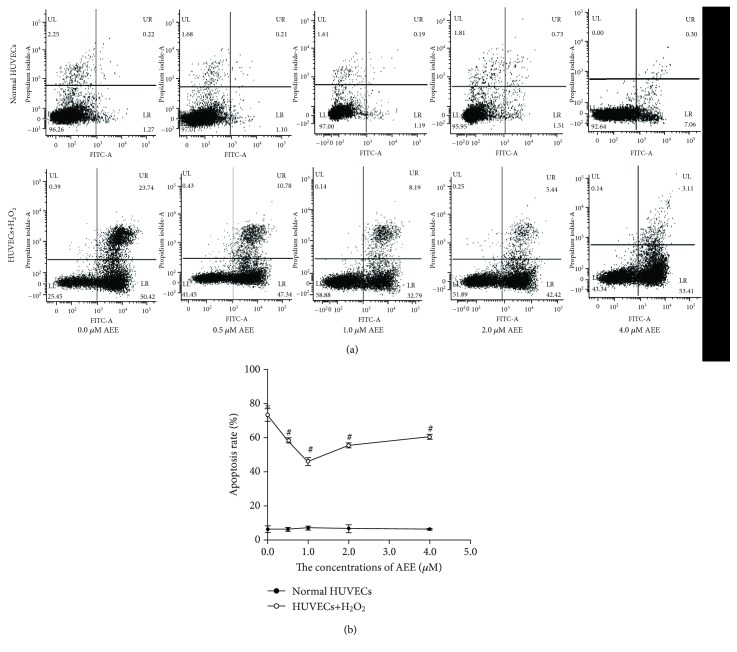
AEE reduced the apoptosis of HUVECs induced by H_2_O_2_. (a) The double stained with Annexin V and PI results among different treatment groups. (b) The apoptosis rate among different treatment groups. Values were expressed as mean ± SD, *n* = 6; ^∗^*p* < 0.05, compared with the normal group; ^#^*p* < 0.05, compared with the group treated with H_2_O_2_ alone (one-way ANOVA followed with Duncan's multiple comparisons).

To verify the effects of AEE on apoptosis in HUVECs, cells were incubated with AEE at 0.5, 1.0, 2.0, and 4.0 *μ*M for 24 h in the absence of H_2_O_2_. As shown in Figure 1, different concentrations of AEE did not cause the apoptosis in HUVECs. Treatment of HUVECs with 300 *μ*M H_2_O_2_ for 6 h significantly increased the apoptosis rate of HUVECs. Reincubating with AEE (0.5, 1.0, 2.0, and 4.0 *μ*M) significantly attenuated H_2_O_2_-induced apoptosis in HUVECs (Figure 1).

### 3.2. AEE Decreased Lipid Peroxidation and Enhanced Antioxidant Ability in the HUVECs

**Figure 2:**
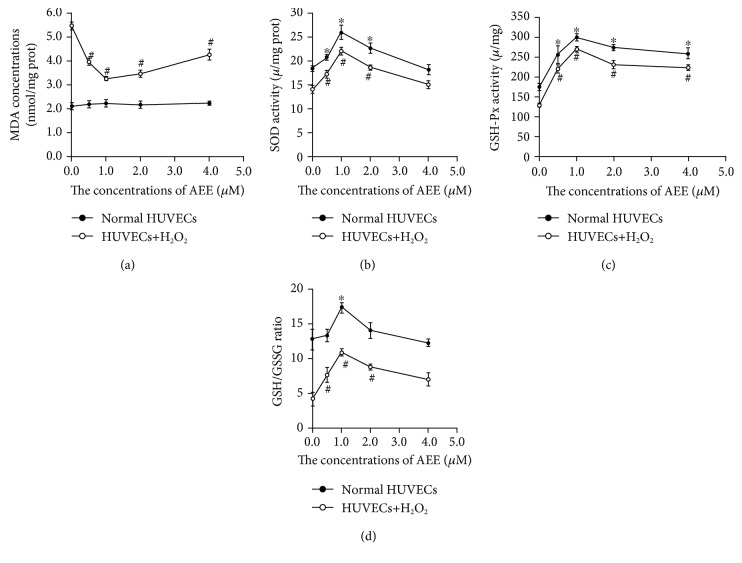
AEE enhanced the antioxidant capacity of HUVECs. (a) AEE decreased lipid peroxidation in the HUVECs. (b) AEE enhanced the SOD activity. (c) AEE raised the GSH-Px activity. (d) AEE increased the ratio of GSH to GSSG. Values were expressed as mean ± SD, *n* = 6; ^∗^*p* < 0.05, compared with the normal group; ^#^*p* < 0.05, compared with the group treated with H_2_O_2_ alone (one-way ANOVA followed with Duncan's multiple comparisons).

The levels of MDA were not different in the HUVECs incubated with different concentrations of AEE (0, 0.5, 1.0, 2.0, and 4.0 *μ*M) for 24 h in the absence of H_2_O_2_. Treatment of HUVECs at 300 *μ*M of H_2_O_2_ for 6 h significantly increased the levels of MDA. However, in the cells pretreated with AEE (0.5, 0.5, 1.0, 2.0, and 4.0 *μ*M), the stimulatory effect of H_2_O_2_ on MDA was significantly reduced (Figure 2(a)).

After the HUVECs were incubated with different concentrations of AEE (0.5, 1.0, 2.0, and 4.0 *μ*M) for 24 h in the absence of H_2_O_2_, the GSH/GSSG ratio and the activity of SOD and GSH-Px were significantly increased. Preincubation with different concentrations of AEE significantly attenuated the decrease in the GSH/GSSG ratio and the activity of SOD and GSH-Px induced by 300 *μ*M of H_2_O_2_ (Figures 2(b)–2(d)).

### 3.3. AEE Ameliorated Lysosomal Disorder Induced by H_2_O_2_

**Figure 3:**
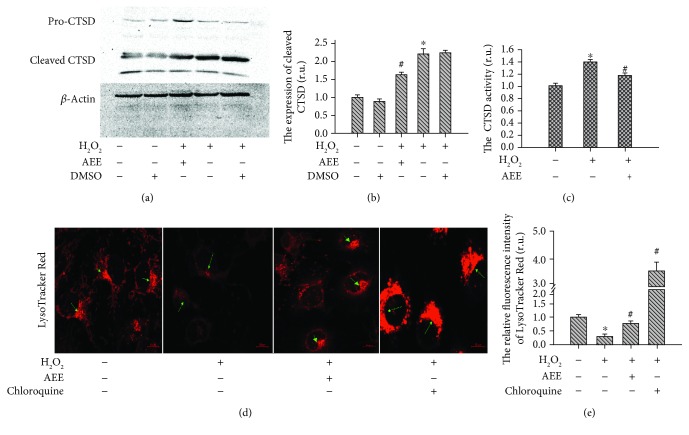
AEE ameliorated lysosomal disorder induced by H_2_O_2_. (a, b) AEE reduced the expression of mature CTSD induced by H_2_O_2_. (c) AEE reduced the increase in CTSD activity induced by H_2_O_2_. The high-density fluorescence of LysoTracker Red was indicated by green arrows. (d, e) AEE enhanced the stability of the lysosomal membrane disrupted by H_2_O_2_ ((d) 10 × 40 power). “+” including the treatment in the HUVECs; “-”excluding the treatment on the HUVECs. Values are expressed as mean ± SD, *n* = 6; ^∗^*p* < 0.05, compared with the normal group; ^#^*p* < 0.05, compared with the group of H_2_O_2_ alone (one-way ANOVA followed with Duncan's multiple comparisons).

The expression and activity of CTSD were examined with Western blotting and enzyme activity test kit. In the control group, the expression of mature CTSD and the activity of CTSD were very low. After treatment with 300 *μ*M H_2_O_2_ for 6 h, the mature CTSD and the activity of CTSD significantly increased. Pretreating HUVECs with 1 *μ*M AEE significantly ameliorated lysosomal disorder manifested as the increased CTSD activity and mature CTSD induced by H_2_O_2_ (Figures 3(a)–3(c)). The lysosomal membrane stability was measured with LysoTracker Red. The LysoTracker Red is an acidophilic dye, which can specifically target to the lysosome, and the LysoTracker fluorescence intensity depends on the acidity of the lysosome. After treatment of the cells with 300 *μ*M H_2_O_2_ for 6 h, the LysoTracker fluorescence intensity significantly decreased compared with the normal group. Pretreating the HUVECs with 1 *μ*M AEE significantly mitigated the decrease in LysoTracker fluorescence intensity induced by H_2_O_2_ (Figures 3(d) and 3(e)).

### 3.4. AEE Mitigated Mitochondrial Dysfunction Induced by H_2_O_2_

**Figure 4:**
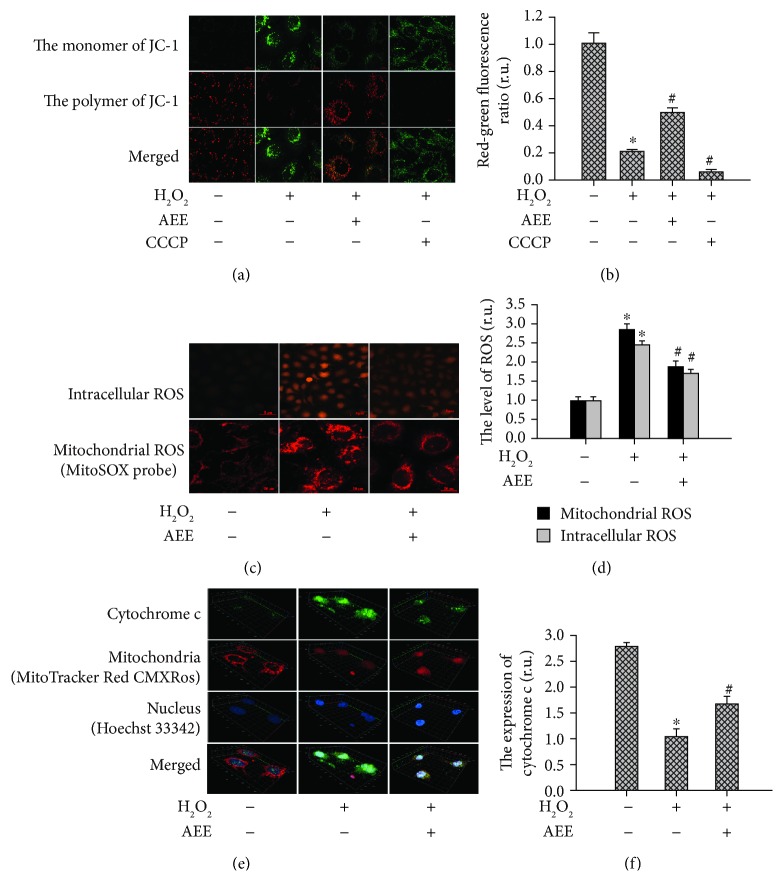
AEE alleviated the mitochondrial dysfunction induced by H_2_O_2_. (a, b) AEE reduced the collapse of the mitochondrial membrane potential induced by H_2_O_2_ ((a) 10 × 40 power). (c, d) AEE reduced the generation of cellular and mitochondrial ROS induced by H_2_O_2_ (upper part of (c): 10 × 10 power; lower part of (c): 10 × 40 power); “+” with the treatment in the HUVECs, “-” excluding the treatment on the HUVECs. (e, f) AEE prevented the release of Cyt c from mitochondria ((e) 10 × 40 power). Values are expressed as mean ± SD, *n* = 6; ^∗^*p* < 0.05, compared with the normal group; ^#^*p* < 0.05, compared with the group of H_2_O_2_ alone (one-way ANOVA followed with Duncan's multiple comparisons).

A JC-1 probe was used to examine the mitochondrial membrane potential. As presented in Figures 4(a) and 4(b), the JC-1 mainly showed the red fluorescence in the control group and mainly showed the green fluorescence in the positive control group and after treatment with 300 *μ*M H_2_O_2_ for 6 h, the ratio of red to green fluorescence intensity significantly decreased compared with the control group, while in the HUVECs pretreated with 1 *μ*M AEE, the mitochondrial membrane potential was significantly higher than in the cells receiving H_2_O_2_ induction alone (*p* < 0.05), indicating that the inhibitory effect of H_2_O_2_ was significantly mitigated by the AEE treatment. The cellular and mitochondrial ROS were assayed with the corresponding fluorescence probe, respectively. The fluorescence intensity for ROS in HUVECs between different treatment groups was showed in Figures 4(c) and 4(d). This illustrated that in the HUVECs preincubated with 1 *μ*M AEE, the increase in cellular and mitochondrial ROS induced by H_2_O_2_ was significantly suppressed. The changes in mitochondrial cytochrome c were investigated via immunofluorescence. The mitochondria were labeled with the MitoTracker Red CMXRos probe (Figure 4(e)). In the control group, the cytochrome c is predominantly located in the mitochondria. However, after the HUVECs were treated with 300 *μ*M H_2_O_2_, the cytochrome c distributing outside the mitochondria increased significantly. In the HUVECs pretreated with 1 *μ*M AEE, the release of cytochrome c from mitochondria induced by H_2_O_2_ was significantly reduced (Figure 4(f)). In short, 300 *μ*M H_2_O_2_ inductions caused mitochondrial dysfunction behaved as collapsed mitochondrial membrane potential, increased ROS production, and release of cytochrome c from mitochondria and those dysfunctions were significantly mitigated by AEE treatment.

### 3.5. AEE Prevented H_2_O_2_-Induced Mitochondrial and Lysosomal Dysfunction via Regulating the Bcl2 Family

#### 3.5.1. AEE Reduced the Changes in Proapoptotic and Antiapoptotic Proteins Induced by H_2_O_2_

**Figure 5:**
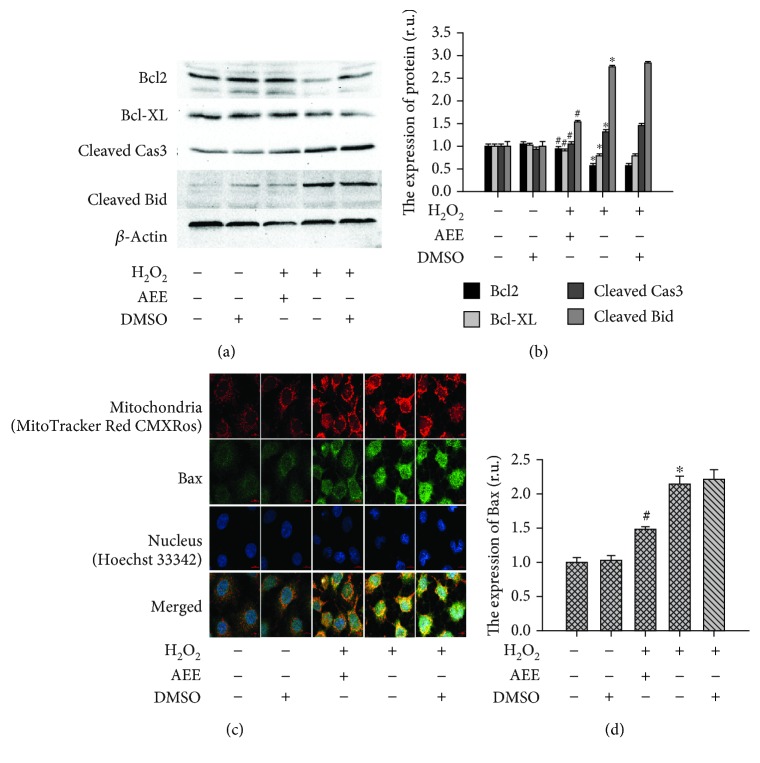
AEE treatment reduced the effect of H_2_O_2_ on antiapoptosis or proapoptosis proteins. (a, b) AEE reduced H_2_O_2_-induced expression of mature Cas3 and Bid and reversed the H_2_O_2_-inhibited expression of Bcl2 and Bcl-XL. (c, d) AEE reduced H_2_O_2_-induced expression of Bax ((c) 10 × 40 power). “+” with the treatment in the HUVECs; “-” without the treatment in the HUVECs. Values are expressed as mean ± SD, *n* = 6; ^∗^*p* < 0.05, compared with the normal group; ^#^*p* < 0.05, compared with the group of H_2_O_2_ alone.

The expression of Bcl2, Bcl-xl, Bax, and Bid was examined with Western blotting and immunofluorescence. In the H_2_O_2_-treated HUVECs, the expression of proapoptotic proteins (Bax and activated Bid) was significantly upregulated while the antiapoptotic proteins (Bcl2 and Bcl-xl) were significantly downregulated. Those changes were reversed by pretreating HUVECs with 1 *μ*M AEE. The apoptotic executioner Cas3 was activated in the H_2_O_2_-treated HUVECs. After treatment with 300 *μ*M H_2_O_2_ for 6 h, the mature Cas3 and its activity were significantly upregulated. Preincubating HUVECs with 1 *μ*M AEE reduced the changes in Cas3 induced by H_2_O_2_ (Figures 5(a)–5(d)).

#### 3.5.2. Genetic Inhibition of Bcl2 Reduced the Effect of AEE on H_2_O_2_-Induced Mitochondrial and Lysosomal Dysfunction

**Figure 6:**
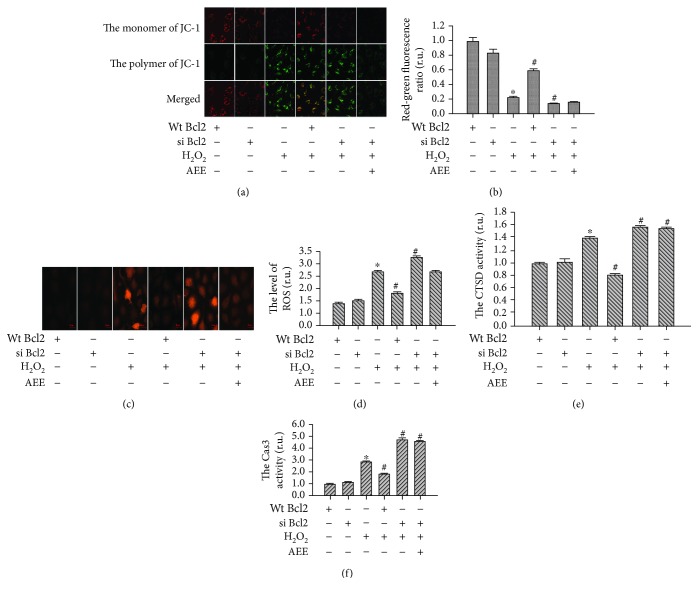
Genetic inhibition of Bcl2 reduced the effect of AEE on H_2_O_2_-induced mitochondrial and lysosomal dysfunction. (a, b) The changes in the mitochondrial membrane potential between different treatment groups ((a) 10 × 40 power). (c, d) The changes in ROS production between different treatment groups ((c) 10 × 40 power). (e, f) The changes in CTSD and Cas3 activity among different treatment groups. “+” with the treatment in the HUVECs; “-” without the treatment in the HUVECs. Values are expressed as mean ± SD, *n* = 6; ^∗^*p* < 0.05, compared with the group of overexpression alone; ^#^*p* < 0.05, compared with the group of H_2_O_2_ alone (one-way ANOVA followed with Duncan's multiple comparisons).

The roles of Bcl2 in the protective effect of AEE on H_2_O_2_-induced mitochondrial and lysosomal dysfunction were investigated. The lentivirus labeled with ubiquitin IRES-puromycin containing siRNA oligonucleotides was used to silence Bcl2 expression. There were over 85% of positive HUVECs transfected with the lentivirus at 5 MOI (multiplicity of infection). The Bcl2 expression was significantly downregulated in the positive HUVECs compared with normal HUVECs. After the HUVECs with downregulated Bcl2 were given 300 *μ*M H_2_O_2_ for 6 h, the mitochondrial membrane potential and lysosomal membrane stability significantly decreased while the ROS generation and the activity of Cas3 and CTSD significantly increased compared with those in the normal HUVECs induced by H_2_O_2_. Preincubating the Bcl2-inhibited HUVECs with AEE did not significantly reverse the changes induced by H_2_O_2_ (Figures 6(a)–6(f)).

#### 3.5.3. Overexpression of Bcl2 Reduced the Mitochondrial and Lysosomal Dysfunction Induced by H_2_O_2_

To confirm the role of Bcl2 in the mitochondrial lysosomal dysfunction induced by H_2_O_2_, HUVECs were transduced with lentivirus to overexpress Bcl2. Bcl2 overexpression significantly ameliorated mitochondrial and lysosomal disorders manifested as the increase in ROS generation and activity of Cas3 and CTSD induced by H_2_O_2_ (Figures 6(a)–6(f)).

## 4. Discussion

Vascular endothelial cells provide a haemocompatible vessel lining via regulating procoagulant and anticoagulant balance of endothelium [33]. The oxidative stress in vessel endothelium would disrupt the balance between anticoagulation and procoagulation to cause cardiovascular disorders [33]. In the present study, vessel endothelium played an important role in the processes of H_2_O_2_-induced oxidative stress in the HUVECs. Our findings confirmed that the incubation of HUVECs with 300 *μ*M H_2_O_2_ for 6 h caused oxidative stress, indicated by the unbalance between antioxidation and oxidation and the dysfunction of mitochondria and lysosome. To evaluate the oxidative stress of cells, many markers have been reported [34–36]. MDA is one of the end products of lipid peroxidation, which is recognized as a sentinel of the cellular oxidation status [36]. In the present study, the levels of MDA enhanced by H_2_O_2_ induction were reduced in the AEE—preincubated HUVECs—and the optimal dose of AEE was found to be 1 *μ*M. The dysfunction of the antioxidant system is responsible for the generation of oxidative stress markers [34]. In the present study, the GSH/GSSG ratio and the activity of SOD and GSH-Px were altered by H_2_O_2_, which was consistent with the previous studies [37, 38]. Pretreatment of the HUVECs with AEE increased the GSH/GSSG ratio and the activity of SOD and GSH-Px and also mitigated the disruption of the antioxidant system induced by H_2_O_2_, suggesting that AEE possessed a good antioxidant activity. This agreed with the previous *in vivo* study that AEE was potential antioxidant. It has been well known that oxidative stress is one of the causes of apoptosis [39, 40]. In the present study, the incubation of the HUVECs with 300 *μ*M H_2_O_2_ for 6 h caused apoptosis and it was significantly reduced by pretreating HUVECs with AEE. This suggests that apoptosis in the HUVECs be related with the unbalance of the oxidative status induced by H_2_O_2_.

Various events involved in oxidative stress and apoptosis are closely related with mitochondrial dysfunction, including generation of ROS, cellular unbalance of the redox status, changes in electron transport and mitochondrial transmembrane potential, release of apoptosis activators (such as cytochrome c), changes in the pro- and antiapoptotic Bcl-2 family proteins, and activation of downstream caspase family proteins [28, 41, 42]. Consistent with these previous studies, our study showed that in the HUVECs with H_2_O_2_-induced oxidative stress there were various characteristics of the oxidative stress related with mitochondrial dysfunction, including increased ROS production, collapsed mitochondrial transmembrane potential, the release of cytochrome c from mitochondria, upregulated proapoptosis Bax and activated Bid, downregulated antiapoptosis Bcl2, and enhanced activity of Cas3. The H_2_O_2_-induced mitochondrial dysfunction was significantly reduced by AEE pretreatment in the HUVECs. In addition to the mitochondrial dysfunctions, the lysosomal dysfunction featured by the destruction of the lysosomal membrane and the increase in CTSD activity were observed in the HUVECs with H_2_O_2_-induced oxidative stress. Again, AEE pretreatment in the HUVECs attenuated the CTSD activity and stabilized the lysosomal membrane. The roles of lysosomes in apoptosis and autophagy have been paid attention, and it has been reported that the lysosomal membrane was destructed by oxidative stress causing the release of CTSD from the lysosome, which activated caspases, Bid, and other potential targets responsible for apoptosis [31, 43–45]. In the present study, we speculated that the increased activation of Bid might be related with the increase in CTSD. Recently, studies on the mutual regulation of mitochondria and lysosome in the apoptosis have confirmed the “lysosomal-mitochondrial axis” theory [32, 46].

To investigate the role of Bcl2 in the protective effect of AEE on H_2_O_2_-induced oxidative stress and lysosomal-mitochondrial axis of apoptosis in the HUVECs, the Bcl2 was knocked down or overexpressed by genetic intervention of Bcl2 proteins and this showed alteration in a variety of mitochondrial events, including mitochondrial transmembrane potential and release of proapoptotic and antiapoptotic factor from mitochondria. Bcl2 proteins inhibited caspase-dependent apoptosis; however, there were few reports showing influence of Bcl2 in lysosomal function in the apoptosis. In the present study, we demonstrated that Bcl2 overexpression significantly reduced H_2_O_2_-induced dysfunctions in mitochondria and lysosome systems, including increase in mitochondrial transmembrane potential and stabilizing lysosomal membrane and decrease in CTSD and Cas3 activity. The knockdown of Bcl2 did not cause the apoptosis in HUVECs but enhanced the mitochondrial and lysosomal dysfunctions induced by H_2_O_2_. It is interesting to note that those changes were not reversed by pretreating HUVECs with AEE. These findings suggested that Bcl2 play a vital role in the protective effect of AEE on H_2_O_2_-induced oxidative stress and apoptosis in the lysosomal-mitochondrial axis in HUVECs. Previous studies have confirmed that AEE has antithrombotic and antiatherosclerotic effects [26, 27]. The metabolomics studies suggested that the antiatherosclerotic effects of AEE might be related with its property of antioxidative stress [26]. In this study, the antioxidative stress effect of AEE was further confirmed *in vitro*. It is worth noticing that the findings of this study suggest that Bcl2 be an important regulation target of AEE to protect cells from oxidative stress. It has been believed that lysosome and mitochondria play important roles in the development of oxidative stress. Our study confirmed that the oxidative stress induced by H_2_O_2_ was involved with the regulation of the lysosomal-mitochondrial axis. However, the interdependent relationships between lysosome and mitochondria are still unclear and further study is required to investigate the mechanisms.

## 5. Conclusion

Our study demonstrated that AEE treatment significantly reduced H_2_O_2_-induced oxidative stress in HUVECs via mitochondria-lysosome axis and Bcl2 was an important regulation target of AEE to protect cells from oxidative stress.

## Data Availability

The data used to support the findings of this study are included within the article.
